# 1-Bromo-4-methyl-2-nitro­benzene

**DOI:** 10.1107/S1600536811036439

**Published:** 2011-09-14

**Authors:** Ping Li, Hai Wang, XiMan Zhang, HongYu Chen

**Affiliations:** a School of Chemistry and Chemical Engineering, TaiShan Medical University, Tai’an 271016, People’s Republic of China

## Abstract

In the title compound, C_7_H_6_BrNO_2_, the dihedral angle between the nitro group and the phenyl ring is 14.9 (11)°.

## Related literature

For related structures, see: Ellena *et al.* (1996 ▶); Gatilov *et al.* (1975 ▶); Fricke *et al.* (2002 ▶). The title compound is an inter­mediate in the synthesis of a pyrethroid insecticide, see: Zou *et al.* (2002 ▶). For the synthesis, see: Moodie *et al.* (1976 ▶).
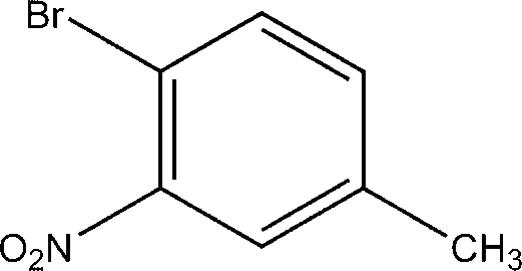


## Experimental

### 

#### Crystal data

C_7_H_6_BrNO_2_
*M* *_r_* = 216.04
Orthorhombic, 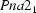

*a* = 13.016 (5) Å
*b* = 14.617 (5) Å
*c* = 4.037 (5) Å
*V* = 768.1 (10) Å^3^
*Z* = 4
Mo *K*α radiation
μ = 5.30 mm^−1^
*T* = 181 K
0.16 × 0.12 × 0.10 mm

#### Data collection

Oxford Diffraction  CCD area-detector diffractometer
Absorption correction: multi-scan (*CrysAlis PRO*; Oxford Diffraction, 2010 ▶) *T*_min_ = 0.627, *T*_max_ = 0.690
3749 measured reflections
1446 independent reflections
1189 reflections with *I* > 2σ(*I*)
*R*_int_ = 0.042

#### Refinement

*R*[*F* ^2^ > 2σ(*F* ^2^)] = 0.053
*wR*(*F* ^2^) = 0.131
*S* = 1.19
1446 reflections
102 parameters
25 restraints
H-atom parameters constrained
Δρ_max_ = 0.85 e Å^−3^
Δρ_min_ = −0.45 e Å^−3^
Absolute structure: Flack (1983 ▶), 556 Friedel pairs
Flack parameter: −0.04 (4)

### 

Data collection: *CrysAlis PRO* (Oxford Diffraction, 2010 ▶); cell refinement: *CrysAlis PRO*; data reduction: *CrysAlis PRO*; program(s) used to solve structure: *SIR97* (Altomare *et al.*, 1999 ▶); program(s) used to refine structure: *SHELXL97* (Sheldrick, 2008 ▶); molecular graphics: *SHELXTL*; software used to prepare material for publication: *WinGX* (Farrugia, 1999 ▶).

## Supplementary Material

Crystal structure: contains datablock(s) global, I. DOI: 10.1107/S1600536811036439/vm2119sup1.cif

Structure factors: contains datablock(s) I. DOI: 10.1107/S1600536811036439/vm2119Isup2.hkl

Supplementary material file. DOI: 10.1107/S1600536811036439/vm2119Isup3.cml

Additional supplementary materials:  crystallographic information; 3D view; checkCIF report

## Figures and Tables

**Fig. 1.**
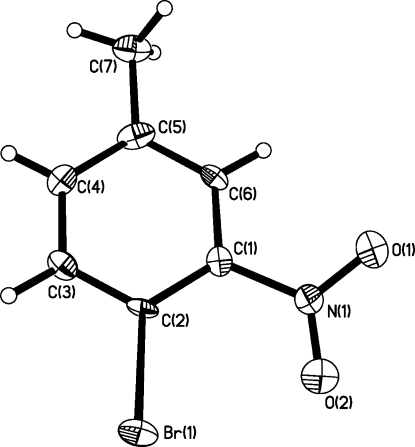
The molecular structure of the title compound with displacement ellipsoids drawn at the 30% probability level.

## supplementary crystallographic information

### Comment

The title compound is a synthetic intermediate in the synthesis of 4-methoxymethylbenzyl alcohol containing bromine, which is an alcohol moiety having insecticidal activity of pyrethroids (Zou *et al.*, 2002). It is a pale yellow liquid, but needle-like crystals were obtained by a slow cooling process from room temperature to 0 °C and the crystal structure was determined at 181 K (Fig. 1).

The dihedral angle between the plane of the nitro group and the best plane through the phenyl ring is 14.9 (11)°. In nitrobenzene structures, the dihedral angle between the nitro group and the phenyl ring is sensitive to its chemial environment, especially the ortho group. In the crystal structure of 4-methyl-2-nitroaniline (Ellena *et al.*,1996), the nitro group having an amino group as neighbour is almost coplanar with the phenyl ring [dihedral angle 3.2 (3)°]. With larger methyl groups as neighbour in pentamethylnitrobenzene (Gatilov *et al.*,1975) the dihedral angle is 86.1 (5)°. In the crystal structure of the analogous compound 2-bromo-3-nitrotoluene (Fricke *et al.*,2002), the dihedral angle between the nitro group and the phenyl ring is 54.1 (4)°.

There are no obvious interactions between neighbouring molecules in the packing.

### Experimental

The title compound was synthesised as described by Moodie *et al.* (1976). The obtained compound is a pale yellow liquid at room temperature. The needle-like crystal was obtained by slowly cooling from room temperature to 0 °C.

### Refinement

All H atoms were geometrically fixed and allowed to ride on their attached atoms, with C-H = 0.93Å for the phenyl group and U_iso_(H)= 1.2U_eq_(C) and C-H = 0.96Å for the methyl group and U_iso_(H)= 1.5U_eq_(C). The U_ij_ components of O1 and O2 have been restrained to isotropic behavior and those of the N—O bonds to have the same U_ij_ components.

### Crystal data

**Table:**

C_7_H_6_BrNO_2_	*F*(000) = 424
*M**_r_* = 216.04	*D*_x_ = 1.868 Mg m^−^^3^
Orthorhombic, *P**n**a*2_1_	Mo *K*α radiation, λ = 0.71073 Å
Hall symbol: P 2c -2n	Cell parameters from 1057 reflections
*a* = 13.016 (5) Å	θ = 3.1–28.9°
*b* = 14.617 (5) Å	µ = 5.30 mm^−^^1^
*c* = 4.037 (5) Å	*T* = 181 K
*V* = 768.1 (10) Å^3^	BLOCK, pale yellow
*Z* = 4	0.16 × 0.12 × 0.10 mm

### Data collection

**Table:**

Oxford Diffraction MODEL NAME? CCD area-detector diffractometer	1446 independent reflections
Radiation source: fine-focus sealed tube	1189 reflections with *I* > 2σ(*I*)
graphite	*R*_int_ = 0.042
phi and ω scans	θ_max_ = 26.4°, θ_min_ = 3.1°
Absorption correction: multi-scan (*CrysAlis PRO*; Oxford Diffraction, 2010)	*h* = −13→16
*T*_min_ = 0.627, *T*_max_ = 0.690	*k* = −18→18
3749 measured reflections	*l* = −4→5

### Refinement

**Table:**

Refinement on *F*^2^	Secondary atom site location: difference Fourier map
Least-squares matrix: full	Hydrogen site location: inferred from neighbouring sites
*R*[*F*^2^ > 2σ(*F*^2^)] = 0.053	H-atom parameters constrained
*wR*(*F*^2^) = 0.131	*w* = 1/[σ^2^(*F*_o_^2^) + (0.0631*P*)^2^] where *P* = (*F*_o_^2^ + 2*F*_c_^2^)/3
*S* = 1.19	(Δ/σ)_max_ < 0.001
1446 reflections	Δρ_max_ = 0.85 e Å^−^^3^
102 parameters	Δρ_min_ = −0.45 e Å^−^^3^
25 restraints	Absolute structure: Flack (1983), 556 Friedel pairs
Primary atom site location: structure-invariant direct methods	Flack parameter: −0.04 (4)

### Special details

**Table:**

Geometry. All esds (except the esd in the dihedral angle between two l.s. planes) are estimated using the full covariance matrix. The cell esds are taken into account individually in the estimation of esds in distances, angles and torsion angles; correlations between esds in cell parameters are only used when they are defined by crystal symmetry. An approximate (isotropic) treatment of cell esds is used for estimating esds involving l.s. planes.
Refinement. Refinement of F^2^ against ALL reflections. The weighted R-factor wR and goodness of fit S are based on F^2^, conventional R-factors R are based on F, with F set to zero for negative F^2^. The threshold expression of F^2^ > 2sigma(F^2^) is used only for calculating R-factors(gt) etc. and is not relevant to the choice of reflections for refinement. R-factors based on F^2^ are statistically about twice as large as those based on F, and R- factors based on ALL data will be even larger.

### Fractional atomic coordinates and isotropic or equivalent isotropic displacement parameters (Å^2^)

**Table:**

	*x*	*y*	*z*	*U*_iso_*/*U*_eq_	
Br1	0.38514 (5)	0.46896 (5)	−0.1387 (5)	0.0399 (3)	
C1	0.1871 (6)	0.4507 (5)	0.1963 (18)	0.0252 (17)	
C2	0.2674 (5)	0.4080 (5)	0.0313 (18)	0.0233 (16)	
C3	0.2651 (6)	0.3145 (5)	−0.003 (2)	0.0307 (18)	
H3	0.3182	0.2847	−0.1126	0.037*	
C4	0.1847 (6)	0.2649 (5)	0.1252 (19)	0.0298 (17)	
H4	0.1855	0.2016	0.1034	0.036*	
C5	0.1030 (6)	0.3055 (6)	0.2844 (19)	0.035 (3)	
C6	0.1046 (5)	0.4002 (5)	0.314 (2)	0.026 (2)	
H6	0.0496	0.4301	0.4135	0.032*	
C7	0.0156 (6)	0.2492 (6)	0.422 (2)	0.044 (2)	
H7A	0.0271	0.1857	0.3726	0.067*	
H7B	−0.0478	0.2686	0.3221	0.067*	
H7C	0.0118	0.2575	0.6572	0.067*	
N1	0.1794 (8)	0.5505 (5)	0.2441 (19)	0.046 (2)	
O1	0.1192 (6)	0.5797 (5)	0.451 (2)	0.073 (3)	
O2	0.2367 (7)	0.5997 (5)	0.110 (2)	0.085 (2)	

### Atomic displacement parameters (Å^2^)

**Table:**

	*U*^11^	*U*^22^	*U*^33^	*U*^12^	*U*^13^	*U*^23^
Br1	0.0283 (4)	0.0560 (5)	0.0354 (4)	−0.0095 (3)	0.0030 (6)	0.0041 (6)
C1	0.023 (4)	0.030 (4)	0.022 (4)	0.004 (3)	−0.010 (3)	−0.002 (3)
C2	0.006 (4)	0.040 (4)	0.024 (4)	−0.002 (3)	0.001 (3)	0.008 (3)
C3	0.014 (4)	0.043 (4)	0.034 (4)	0.004 (3)	−0.004 (3)	−0.001 (3)
C4	0.025 (5)	0.030 (4)	0.035 (4)	−0.001 (3)	−0.012 (3)	0.002 (3)
C5	0.020 (4)	0.045 (4)	0.042 (7)	−0.012 (3)	−0.014 (3)	0.014 (4)
C6	0.018 (4)	0.040 (4)	0.021 (6)	0.001 (3)	0.002 (3)	−0.003 (4)
C7	0.044 (5)	0.055 (5)	0.034 (5)	−0.021 (4)	−0.005 (4)	0.001 (4)
N1	0.061 (5)	0.034 (4)	0.043 (4)	0.003 (4)	0.018 (3)	0.000 (3)
O1	0.090 (5)	0.050 (4)	0.079 (6)	0.000 (3)	0.037 (4)	−0.012 (3)
O2	0.099 (5)	0.053 (4)	0.104 (5)	−0.006 (4)	0.053 (5)	−0.003 (4)

### Geometric parameters (Å, °)

**Table:**

Br1—C2	1.901 (7)	C5—C6	1.389 (12)
C1—C6	1.386 (10)	C5—C7	1.510 (10)
C1—C2	1.389 (10)	C6—H6	0.9300
C1—N1	1.475 (10)	C7—H7A	0.9600
C2—C3	1.373 (10)	C7—H7B	0.9600
C3—C4	1.373 (11)	C7—H7C	0.9600
C3—H3	0.9300	N1—O2	1.170 (10)
C4—C5	1.377 (11)	N1—O1	1.222 (10)
C4—H4	0.9300		
			
C6—C1—C2	120.5 (7)	C6—C5—C7	121.5 (8)
C6—C1—N1	115.5 (7)	C1—C6—C5	120.9 (7)
C2—C1—N1	123.9 (7)	C1—C6—H6	119.6
C3—C2—C1	118.6 (7)	C5—C6—H6	119.6
C3—C2—Br1	116.6 (5)	C5—C7—H7A	109.5
C1—C2—Br1	124.7 (5)	C5—C7—H7B	109.5
C4—C3—C2	120.3 (7)	H7A—C7—H7B	109.5
C4—C3—H3	119.9	C5—C7—H7C	109.5
C2—C3—H3	119.9	H7A—C7—H7C	109.5
C3—C4—C5	122.4 (7)	H7B—C7—H7C	109.5
C3—C4—H4	118.8	O2—N1—O1	120.7 (9)
C5—C4—H4	118.8	O2—N1—C1	120.3 (8)
C4—C5—C6	117.2 (7)	O1—N1—C1	118.6 (8)
C4—C5—C7	121.3 (7)		
